# GranoScan: an AI-powered mobile app for in-field identification of biotic threats of wheat

**DOI:** 10.3389/fpls.2024.1298791

**Published:** 2024-06-07

**Authors:** Riccardo Dainelli, Antonio Bruno, Massimo Martinelli, Davide Moroni, Leandro Rocchi, Silvia Morelli, Emilio Ferrari, Marco Silvestri, Simone Agostinelli, Paolo La Cava, Piero Toscano

**Affiliations:** ^1^ Institute of BioEconomy (IBE), National Research Council (CNR), Firenze, Italy; ^2^ Institute of Information Science and Technologies (ISTI), National Research Council (CNR), Pisa, Italy; ^3^ Barilla G. e R. Fratelli S.p.A., Parma, Italy

**Keywords:** deep learning, in-field recognition, disease, pest, weed, winter cereals, free mobile app, co-design thinking

## Abstract

Capitalizing on the widespread adoption of smartphones among farmers and the application of artificial intelligence in computer vision, a variety of mobile applications have recently emerged in the agricultural domain. This paper introduces GranoScan, a freely available mobile app accessible on major online platforms, specifically designed for the real-time detection and identification of over 80 threats affecting wheat in the Mediterranean region. Developed through a co-design methodology involving direct collaboration with Italian farmers, this participatory approach resulted in an app featuring: (i) a graphical interface optimized for diverse in-field lighting conditions, (ii) a user-friendly interface allowing swift selection from a predefined menu, (iii) operability even in low or no connectivity, (iv) a straightforward operational guide, and (v) the ability to specify an area of interest in the photo for targeted threat identification. Underpinning GranoScan is a deep learning architecture named efficient minimal adaptive ensembling that was used to obtain accurate and robust artificial intelligence models. The method is based on an ensembling strategy that uses as core models two instances of the EfficientNet-b0 architecture, selected through the weighted F1-score. In this phase a very good precision is reached with peaks of 100% for pests, as well as in leaf damage and root disease tasks, and in some classes of spike and stem disease tasks. For weeds in the post-germination phase, the precision values range between 80% and 100%, while 100% is reached in all the classes for pre-flowering weeds, except one. Regarding recognition accuracy towards end-users in-field photos, GranoScan achieved good performances, with a mean accuracy of 77% and 95% for leaf diseases and for spike, stem and root diseases, respectively. Pests gained an accuracy of up to 94%, while for weeds the app shows a great ability (100% accuracy) in recognizing whether the target weed is a dicot or monocot and 60% accuracy for distinguishing species in both the post-germination and pre-flowering stage. Our precision and accuracy results conform to or outperform those of other studies deploying artificial intelligence models on mobile devices, confirming that GranoScan is a valuable tool also in challenging outdoor conditions.

## 1 Introduction

From cell to farm level, scientific advances have always led to a better understanding of how various components of the agricultural system interact (Jung et al., 2021). This is particularly true in the current challenging period, including the global pandemic, supply chain breakdowns, drought-driven by climate change, and war, where precision agriculture needs to face increasing pressure for resource availability in combination with the projected increase in food demand by more than 70% by 2050 (World Bank Group, 2023). For agricultural optimization, emerging technologies, such as big data analysis, the internet of things (IoT), geospatial technologies and artificial intelligence (AI), are promising tools aimed at enhancing crop production and reducing inputs (Sishodia et al., 2020). AI proposes important contributions to knowledge pattern classification as well as model identification that might solve issues in the agricultural domain (Lezoche et al., 2020). Computer vision has been utilized to provide accurate, site-specific information about crops and their environments (Lu and Young, 2020).

The history of computer vision applied to the agri-food chain started in the mid-1980s, mainly with seed and fruit sorting (Berlage et al., 1984; Rehkugler and Throop, 1986) and plant identification (Guyer et al., 1986). However, the explosion of agricultural computer vision took place at the beginning of the 2010s, with more than 2000 research papers published per year (Web of Science, 2023), thanks to reduced equipment costs and increased computational power (Patrício and Rieder, 2018). In the 2010s, highly cited papers reported several applications of computer vision for in-field plant identification (Grinblat et al., 2016; Hamuda et al., 2016; Jin et al., 2017; Tenhunen et al., 2019), plant phenotyping (Fahlgren et al., 2015; Ubbens and Stavness, 2017; Virlet et al., 2017; Ghosal et al., 2018), fruit counting and quantity and quality evaluation (Cubero et al., 2011; Rahnemoonfar and Sheppard, 2017; Ponce et al., 2019; Yu et al., 2019). Since 2020, the previous agricultural themes have been developed through many herbaceous and tree crops considering robotics (Fu et al., 2020; Wu et al., 2020), advanced deep learning (DL) techniques (da Costa et al., 2020; Santos et al., 2020; Miragaia et al., 2021), and various real environments (Fonteijn et al., 2021; Borraz-Martínez et al., 2022). Other new aspects are addressed such as crop type mapping (Nowakowski et al., 2021), soil organic matter prediction (Taneja et al., 2021), nutrient content/demand determination (Iatrou et al., 2021; Ahsan et al., 2022) or abiotic stress monitoring (Azimi et al., 2021; Zermas et al., 2021; Kumar et al., 2022). In addition, researchers paid particular attention to the pivotal and challenging issue of in-field localization and recognition of pests (Høye et al., 2021; Wang et al., 2021), diseases (Su et al., 2020; Nagaraju et al., 2022) and weeds (de Castro et al., 2018; Gallo et al., 2023). Regarding wheat crop, the most recent scientific works deal with spike segmentation and counting (David et al., 2020; Ma et al., 2020; Misra et al., 2022), leaf (Bao et al., 2021) and spike (Su et al., 2020) disease identification and post-harvest grain quality monitoring (AgaAzizi et al., 2021; He et al., 2021; Zhao et al., 2022).

Applications of the computer vision system in agriculture are promising in unraveling different problems (Patrício and Rieder, 2018). They raise productivity, by automating laborious tasks in a non-destructive way, improve quality and ultimately increase the profitability of farmers and other stakeholders (Meshram et al., 2021). Nevertheless, open issues still remain to be solved. Considering that computer vision systems leverage AI and especially machine learning (ML), the availability of high-quality data for training these architectures plays a crucial role. In this sense, the preparation of agricultural image datasets is strenuous because of the efforts and costs required for image acquisition, categorization and annotation. Most of the currently published datasets have several limitations, such as the small number of samples and image collection in a non-field environment, without addressing the complexity of open fields (Wang et al., 2021). In addition, although sharing saves significant resources and enables benchmarking of image analysis and machine learning algorithms (Lobet, 2017), the datasets publicly available are few (Orka et al., 2023). As a case study, Lu and Young (Lu and Young, 2020) in their survey retrieved 5870 search records, but only 34 datasets complied with the inclusion criteria of public availability (no need for a request to the authors) and image collection in field or quasi-field conditions. Besides, despite there being many general and open-source software libraries and toolkits, such as OpenCV (OpenCV, 2023), TensorFlow (TensorFlow, 2023), PyTorch (PyTorch, 2023), scikit-learn (Scikit-learn, 2023), open-source and end-to-end platforms that develop computer vision systems for the agricultural domain are not so numerous. In brief, we report three examples: AirSurf, an automated and open-source analytic platform to measure yield-related phenotypes from ultra-large aerial imagery (Bauer et al., 2019); CoFly, a modular platform incorporating custom-developed AI and information and communication technologies (ICT) for unmanned aerial vehicle (UAV) applications in precision agriculture (Raptis et al., 2023); and Fiware, a general framework of open-source platform components for developing and integrating also smart farming solutions (Fiware, 2023).

Mobile devices and especially smartphones are an extremely popular source of communication for farmers (Raj et al., 2021). In the last decade, a variety of applications (mobile apps) have been developed according to farmers’ needs (Mendes et al., 2020). Their added value consists of locating all the different information in one place that farmers can directly and intuitively access (Patel and Patel, 2016). The photographic record through the embedded smartphone camera and the interpretation or processing of images is the focus of most of the currently existing applications (Mendes et al., 2020). In particular, agricultural apps deploy computer vision systems to support decision-making at the crop system level, for protection and diagnosis, nutrition and irrigation, canopy management and harvest.

Analyzing technical gaps associated with the development of accurate, reliable and easy-to-use mobile apps for crop diagnosis, the availability of high-quality data for training deep learning architectures remains an actual bottleneck. This is mainly due both to the lack of in-field data and the efforts (time and labor) required to acquire and pre-process images, i.e. reshaping, resizing, categorization, annotation. In addition, due to legal restrictions, data transfer speeds and network issues, the app’s functioning sometimes may be slowed down (Kirk et al., 2011). Regarding issues in using the apps, poor lighting when reading information on small screens, especially in bright field conditions and apps providing too many recommendations with a lack of site-specific information were reported (Thar et al., 2021). Free mobile apps available in digital stores are poorly documented, as the vast majority of apps do not have a supporting peer-reviewed publication. The lack of a solid scientific basis could undermine the reliability of the app, mainly in terms of performance. Other issues in agricultural mobile app development concern social gaps, mostly represented by trust, comfort and affordances in adopting this technology by end users (farmers). The development of a digital tool requires early and ongoing interactions with targeted users to clarify app goals and features, ensure the reliability of scientific input and optimize farmer experience (Inwood and Dale, 2019). Also, training would be beneficial to effectively understand and properly use this type of app. As stated by Thar et al. (Thar et al., 2021), farmers are optimistic about agricultural mobile apps with over 70% of the respondents in their survey willing to use them. The gap arises between the positive attitude toward agricultural mobile apps and the negative usage level of most farmers: this is the real challenge to be tackled.

Regarding crop abiotic and biotic stress recognition and diagnosis, many mobile tools have been implemented so far. They are dedicated both to a set of crops - Leaf Analysis (Petrellis, 2019); E-agree (Reddy et al., 2015); the smart system proposed by Chen et al. (Chen et al., 2020) - and a specific crop. For example, e-RICE categorizes the symptoms to make an accurate diagnosis of common rice diseases and problems (Morco et al., 2017); the TobaccoApp detects any damage on tobacco leaf caused by fungi (Valdez-Morones et al., 2019); AuToDiDAC detects, separates and assesses the diseases in cacao black pod rot (Tan et al., 2018). Nevertheless, free apps available in online stores and supported by a research paper are quite rare. Among those, it is worth mentioning: ApeX−Vigne, which monitors vine water status using crowdsourcing data (Pichon et al., 2021); Plantix, which detects, through deep learning algorithms, diseases, pests, and nutritional deficiencies in 30 crop types (Tibbetts, 2018); BioLeaf, which measures *in situ* foliar damage caused by insects (MaChado et al., 2016); PlantifyAI, for diagnosing 26 diseases across 14 crop species by offering also control methods (Shrimali, 2021); and PlantVillage Nuru, which leverages a crowdsensing platform for plant disease diagnosis in developing countries (Coletta et al., 2022). Within this group, no mobile applications are specifically dedicated to wheat crop.

Within this framework, the current paper presents GranoScan, a free mobile app dedicated to field users. The most common diseases, pests and weeds affecting wheat both in pre and post-tillering were selected. An automatic system based on open AI architectures and fed with images from various sources was then developed to localize and recognize the biotic agents. After cloud processing, the results are instantly visualized and categorized on the smartphone screen, allowing farmers and technicians to manage wheat rightly and timely. In addition, the mobile app provides a disease risk assessment tool and an alert system for the user community. The design and implementation of GranoScan aim to ensure a foolproof detection system and, at the same time, a user-friendly experience.

The main contributions of the current study are highlighted hereafter:

- develop a deep learning architecture for recognizing threats affecting wheat, which leverages images directly acquired in the field with the smartphone camera;
- release a simple-to-use and free smart tool dedicated to farmers and field technicians, implemented through a co-design process together with these stakeholders;
- create a user community capable of promoting good agricultural practices through the use of the GranoScan app.

The paper is structured as follows: Section 2 showcases the app co-design workflow, the selection of threats and the underpinning deep learning architecture. Section 3 and Section 4 describe and discuss, respectively, the results of the co-design process, the app graphic features and the app performances in recognizing wheat abiotic and biotic stresses, also towards users’ real use. Finally, Section 5 summarizes the usefulness of GranoScan, underlining farmer engagement, and gives previews of the app’s future developments.

## 2 Materials and methods

### 2.1 Mobile application co-design (workflow and app design)

Involving potential users in the design of a digital solution is a necessity (Kenny and Regan, 2021). Co-designing activities with farmers for the implementation of a mobile app in agriculture can help ensure that the app meets the needs of its intended users and is effective in providing the expected solutions. Despite restrictions due to the COVID-19 pandemic preventing live meetings, we were able to identify over 40 farmers from different Italian regions who were interested in our project and willing to participate in the design process. Once the group of interested farmers had been identified, we planned monthly online meetings to discuss the app’s purpose and functionality. During these meetings, farmers provided feedback on the features they would like to see in the app and how they would like to use it. This feedback was used to create the first prototype of the app, which was tested and refined through ongoing discussions and feedback from the farmers. This group of farmers was further involved in the app’s prototype promotion, which ensured that a group of over 100 beta testers consisted of farmers. The participatory approach allowed farmers to contribute their knowledge and skills to ensure that the app meets their needs and is user-friendly. Several topics and needs emerged from the discussions: the graphics of the app in terms of colors, icons, and text size to ensure simple use in the field with different light conditions; the request for an easy user-application iteration with a quick selection from a pre-set menu; the possibility of using it even in conditions of poor connectivity or total absence of connection; the ability to handle unknown cases; a quick and simple guide to operating correctly; the option to indicate an area of interest on the photo for which to request recognition; a dedicated section where the results can be consulted at any time; to be informed of any plant diseases found in fields close to their own. Additionally, the 40 farmers, together with technicians, researchers and project partners, were involved in the selection of diseases, pests and weeds.

### 2.2 Disease, pest and weed selection and image retrieval

As for the app functions and graphics, the stakeholders were requested to contribute to the list of the main biotic agents affecting wheat in the Mediterranean environment. Starting from a scientific literature survey, an intense consultation activity involving farmers, technicians and researchers was carried out, allowing the selection of the target diseases, pests and weeds.

Diseases are represented by those caused by a single fungus or a species complex (**Supplementary Table S1**). The detected diseases affect all the organs of wheat (root, leaf, stem, spike). Regarding pests (**Supplementary Table S2**), the focus is mainly on insects but slugs and mites are also included. Insects are recognized in different life cycle stages (egg, larvae, adult). Weeds encompass both monocot and dicot and a species belonging to *Tracheophytes*, i.e. the common horsetail (*Equisetum arvense*) (**Supplementary Table S3**). Weeds are recognized both in the seedling stage (“Biologische Bundesanstalt, Bundessortenamt und CHemische Industrie” (BBCH) scale, stages 10–19) (Meier, 2018) and from nine true leaves onwards. For seven of the most widespread and hard-to-control species, a phenotyping activity was conducted to create an in-house imagery dataset. The selection was made by considering (i) bottom-up information and specific requests by farmers and technicians, (ii) weeds susceptibility <50% to commercial formulations for chemical control as reported at least twice by field technicians, and (iii) hard to control species considering other methods (agronomical, mechanical, etc.). This way, the training of the developed AI architecture can be boosted with low-cost and high-resolution images (see section 2.2.1). In addition to those biotic agents, frost damage on spikes and cereal leaf beetle (*Oulema melanopus*) damage on leaves are also encompassed.

Raw images for training the implemented AI architecture were retrieved from different sources, that is stakeholders of the wheat supply chain and research activities. In the first case, farmers and technicians engaged during co-design anonymously shared raw images taken in the field through a dedicated web application (even during the COVID-19 pandemic). In the second case, researchers carried out field scouting and phenotyping activity.

#### 2.2.1 Weed phenotyping

Phenotyping was conducted both on monocots and dicots (**Supplementary Table S3**), selected through the overall list of weeds recognized by GranoScan. Considering the agronomic relevance of these seven weeds, the phenotyping activity was necessary to enhance the number of images, completing those retrieved from the in-field acquisition. Weed seeds were sown in April and November 2021 in 36 black plastic pots for each species and placed in a growth chamber with optimal microclimatic and agronomic conditions. For image shooting into the open air, a Canon EOS 700D hand-held camera was used. The acquisition was facilitated by using a white panel as a background and performed with homogeneous light conditions (full sunlight/full shade), avoiding mixed situations that could hinder the automatic recognition system. As also suggested by other studies (Wang et al., 2021), photo capture timing, target distances and light conditions did not have a fixed pattern but were deliberately programmed to vary in such a way as to mimic field conditions that a user may experience. The images were acquired until the pre-flowering stage but focus was placed especially on the post-emergence targets (BBCH 10–19) because early identification of weeds allows the control to be more effective. The final phenotyping dataset includes 10810 images and is publicly shared in an open-access repository (Dainelli et al., 2023).

### 2.3 Image dataset processing

The dataset has been divided into nine parts, as in **Table 1**. Each part contains images suited for a specific identification and classification task. For instance, the “Leaf disease” task refers to identifying in the image possible areas interested by disease, e.g. parts of the leaves on which the signs of *Septoria* are visible. The total number of images is 67302. This number is given by the number of original images retrieved from different sources (31335, number of real images, **Table 1**) supplemented by additional images obtained by a data augmentation procedure for leaf disease, leaf damage, spike disease, spike damage, stem disease and root disease tasks (**Table 1**). In this procedure, random rotations, and changes in tone and intensity were applied to obtain variants of the original images, increasing the size of the dataset, excluding pests and weeds tasks, by approximately a factor of 6. Indeed, note that the number is not divisible by 6, since augmented images were used only in the training and validation phases and not in the testing one. Moreover, the augmented images were re-checked manually and visually inspected to remove those in which the transformation had led to underexposed and overexposed images or produced a crop excluding the area of interest.

**Table 1:** The GranoScan dataset.

Task	Classes	Number of real images	Number of images after augmentation
Leaf disease	6: healthy, *Blumeria graminis f.* sp. *tritici, Puccinia recondita f.* sp. *tritici, Puccinia striiformis f.* sp. *tritici, Puccinia graminis f.* sp. *tritici, Septoria tritici*	5345	12259
Leaf damage	2: healthy, damaged by *Oulema melanopus*	910	11027
Spike disease	5: healthy, *Fusarium graminearum, Blumeria graminis f.* sp. *tritici, Puccinia graminis f.* sp. *tritici, Stagonospora nodorum*	831	9009
Spike damage	2: healthy, damaged by frost	326	4651
Stem disease	3: healthy, *Blumeria graminis f.* sp. *tritici*, *Puccinia graminis f.*sp*.tritici*,	468	6475
Root disease	2: healthy, root rot (*Gaeumannomyces graminis*, *Fusarium* spp., *Bipolaris sorokiniana*)	32	458
Pests	36: *Oulema melanopus* (larvae, adult), *Haplodiplosis marginata* (larvae, adult), *Mayetiola destructor* (larvae, adult), *Contarina tritici* (larvae, adult), *Sitodiplosis mosellana* (larvae, adult), *Eurygaster maura* (egg, adult), *Aelia rostrata* (egg, adult), *Sitobion avenae* (larvae, adult), *Rhopalosiphum padi* (larvae, adult), *Agriotes* (larvae, adult), *Chlorops pumilionis* (larvae, adult), *Oscinella frit* (larvae, adult), *Delia coarctata* (larvae, adult), *Dermaptera* (adult), *Myriapoda* (adult), *Carabidae*-*Chrysomelidae*-*Curculionidae* (adult), *Noctua* (adult), *Arachnida* (adult), *Arion*-*Deroceras*-*Limax* (adult), *Coccinella* (egg, larvae, pupa and adult)	3622	3622
Post-germination weeds	36: *Alopecurus myosuroides*, *Anthemis arvensis*, *Apera spica-venti*, *Avena sterilis*, *Bifora radians*, *Brassica rapa subs. oleifera*, *Capsella bursa-pastoris*, *Centaurea cyanus*, *Cerastium holosteoides*, *Cirsium arvense*, *Convolvulus arvensis*, *Convolvulus sepium*, *Equisetum arvense*, *Fallopia convolvulus*, *Fumaria officinalis*, *Galeopsis tetrahit*, *Galium aparine*, *Geranium molle*, *Lamium purpureum*, *Lolium* spp., *Matricaria chamomilla*, *Oxalis* spp., *Papaver rhoeas*, *Phalaris* spp., *Poa annua*, *Poa trivialis*, *Polygonum aviculare*, *Polygonum persicaria*, *Ranunculus arvensis*, *Raphanus raphanistrum*, *Sinapis arvensis*, *Stellaria media*, *Veronica hederifolia*, *Veronica persica*, *Vicia* spp., *Viola* spp.	15010	15010
Pre-flowering weeds	36: same species as post-germination weeds	4791	4791

The datasets were then annotated and conditioned in a task-specific fashion. In particular, in tasks related to pests, weeds and root diseases, for which a deep learning model based on image classification is used, all the images have been cropped to produce square images and then resized to 512x512 pixels. Images were then divided into subfolders corresponding to the classes reported in **Table 1**.

**Figure 1:**
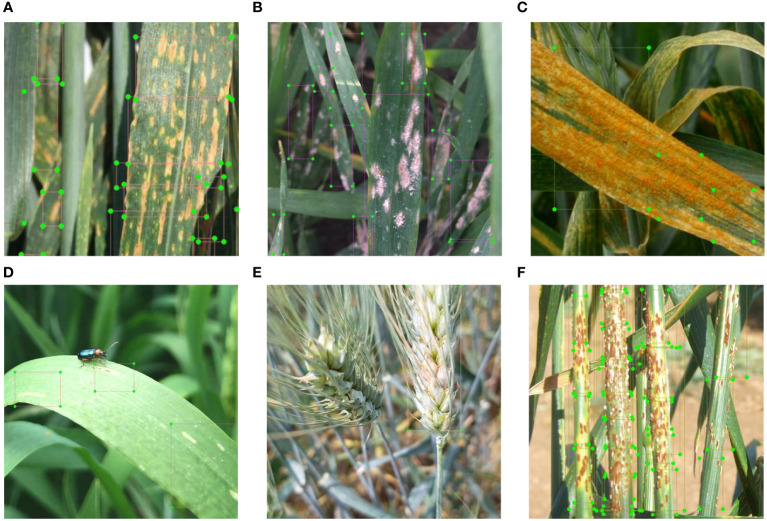
Examples of images manually annotated for object detection tasks: leaf disease – **(A)** septoria; **(B)** powdery mildew; **(C)** yellow rust; leaf damage – **(D)** damage from cereal leaf beetle; spike disease - **(E)** Fusarium head blight; and stem disease – **(F)** black rust.

In all the other tasks, where an object detection model is used, the images were first annotated by manually drawing a set of rectangular areas in which particular diseases or damages are visible (**Figure 1**). Each rectangle is labeled with the classes reported in **Table 1** for a total of 58101 annotations before data augmentation. To this end, the annotation tool LabelImg (Tzutalin, 2015) was used. Afterward, all the images were resized to 256x256 pixels for leaf, spike and stem diseases.

### 2.4 Deep learning architecture

To obtain a classification system for the images we collected, we opted to use an original method that we studied and implemented. More in detail, to get accurate and robust AI models, we used a deep learning architecture named efficient minimal adaptive ensembling that we already tested (Bruno et al., 2022) by setting the new state-of-the-art with an accuracy of 100% on the Plantvillage public dataset. The method is based on an ensembling strategy that uses as core models two instances of the EfficientNet-b0 architecture. More precisely, the EfficientNet family (Tan and Le, 2019) consists of 8 instances, numbered from EfficientNet-b0 to EfficientNet-b7, that have an increasing complexity and number of parameters. All the members of the EfficientNet family have been designed to have efficiency as a target and have been obtained by using a structured method to generate a compound scaling of the network’s depth, width and resolution. According to previous works and experimental evidence (Bruno et al., 2023), the b0 variant of the EfficientNet family fits better with the need for the GranoScan app to provide results with high accuracy and low latency. In addition, instead of using one single instance of trained EfficientNet-b0, we have adopted the ensembling technique, which aims to transform a number of weak models (in the present case, each one represented by a single EfficinetNet-b0) into a strong classifier named “ensemble” model. Ensembling is performed by an innovative strategy of performing bagging at the deep feature level. Namely, only the convolutional layers of each trained weak model are kept, while the final decisional layers are neglected; in this way, each weak model is turned into an extractor of deep features. The deep features of each weak model are then concatenated and fed to a trainable final decision layer (Bruno et al., 2023), to which we refer for more details on the ensembling construction).

The proposed method encompasses eight main design choices: (i) first, data stratification was introduced to cope with unbalanced data and allow improved performances; (ii) transfer learning was used for providing a faster convergence, specifically instances of EfficientNet-b0 networks pre-trained on the ImageNet task were used as initial models; (iii) cross-entropy loss was employed, given the multiclass nature of all the addressed problems and class-imbalance issues; such loss is a natural choice since it exponentially penalizes differences between predicted and true values, expressed as the probability of the class to which they belong; (iv) Adabelief optimizer was selected for faster convergence and better generalization, also (v) making use of regularization to improve robustness to noises; (vi) the weighted F1-score, which takes into account misclassification and unbalanced data, was employed; (vii) ensembling was performed using the minimum number of weak classifiers (that is, two) in a such a way as to improve overall classification performances (as demonstrated experimentally) while limiting complexity; (viii) the resulting ensemble was fine-tuned only, reducing the ensemble training complexity.

The training and validation process for the ensemble model involved dividing each dataset into training, testing, and validation sets with an 80–10-10 ratio. Specifically, we began with end-to-end training of multiple models, using EfficientNet-b0 as the base architecture and leveraging transfer learning. Each model was produced from a training run with various combinations of hyperparameters, such as seed, regularization, interpolation, and learning rate. From the models generated in this way, we selected the two with the highest F1 scores across the test, validation, and training sets to act as the weak models for the ensemble. The original decision layers of these weak models were removed, and a new decision layer was added, using the concatenated outputs of the two weak models as input. This new decision layer was trained and validated on the same training, validation, and test sets while keeping the convolutional layers from the original weak models frozen. Lastly, a fine-tuning process was applied to the entire ensemble model to achieve optimal results.

The ensembling is performed using a linear combination layer that takes as input the concatenation of the features processed by the weak models and returns the linear mapping into the output space. During the fine-tuning, the parameters of the weak models are frozen and the linear layer only is trained. In this way, the resulting ensemble is efficient because the computational costs are very close to the cost of a single model (because only a small fraction of the parameters are updated and, since the weak models are independent, it is possible to parallelize their training) and adaptive (because the layer performing the ensemble is trained on the real data and it is not a mere aggregation function, as commonly used).

For the sake of the reproducibility of the results, further considerations about the architecture and its training are collected. The kernel sizes of the weak models are the standard blocks in the EfficientNet-b0 as reported in the original paper (Tan and Le, 2019). As a rule of thumb, Stride 2 was used for depth convolutional blocks, while Stride 1 was selected for all the other ones. As an activation function borrowed from the EfficientNet family, SiLU (i.e. Sigmoid Linear Unit) was preferred over ReLU (i.e. Rectified Linear Unit). This activation function is a particular case obtained by setting β = 1 in the Swish activation function (Hendrycks and Gimpel, 2016). SiLU inherits two good properties from its more general variant: it is smooth and less sensitive to the vanishing gradients problem with respect to ReLU. In the training procedures, the maximum number of epochs was set to 100. An early stopping mechanism was used and assigned to 10 epochs without improvements (i.e. in technical jargon, the patience was set to 10). Generally, after 18–20 epochs, the models reached their best performance. The learning rate was set to 0.0005 and was not changed during training.

### 2.5 App security and interaction with the deep learning model

Security-related issues are of pivotal importance to guarantee data protection and user privacy. In GranoScan, the authorization filter has been implemented following OAuth2.0-like specifications to guarantee a high-level security standard. All data are transmitted and received in an encrypted way and the resources accessibility is managed by a temporary access token generated by the system and it can be regenerated through a refresh token. To minimize the throughput of requests for tokens management, refresh and access tokens are stored in a specific private area of the mobile app until the time expires.

Regarding the development and deployment of the app, GranoScan follows AgroSat (AgroSat, 2023) APIs specifications and implements Flutter technologies to ease GranoScan porting on Android and iOS devices. The GranoScan app is released and maintained on Google and Apple app stores.

**Figure 2:**
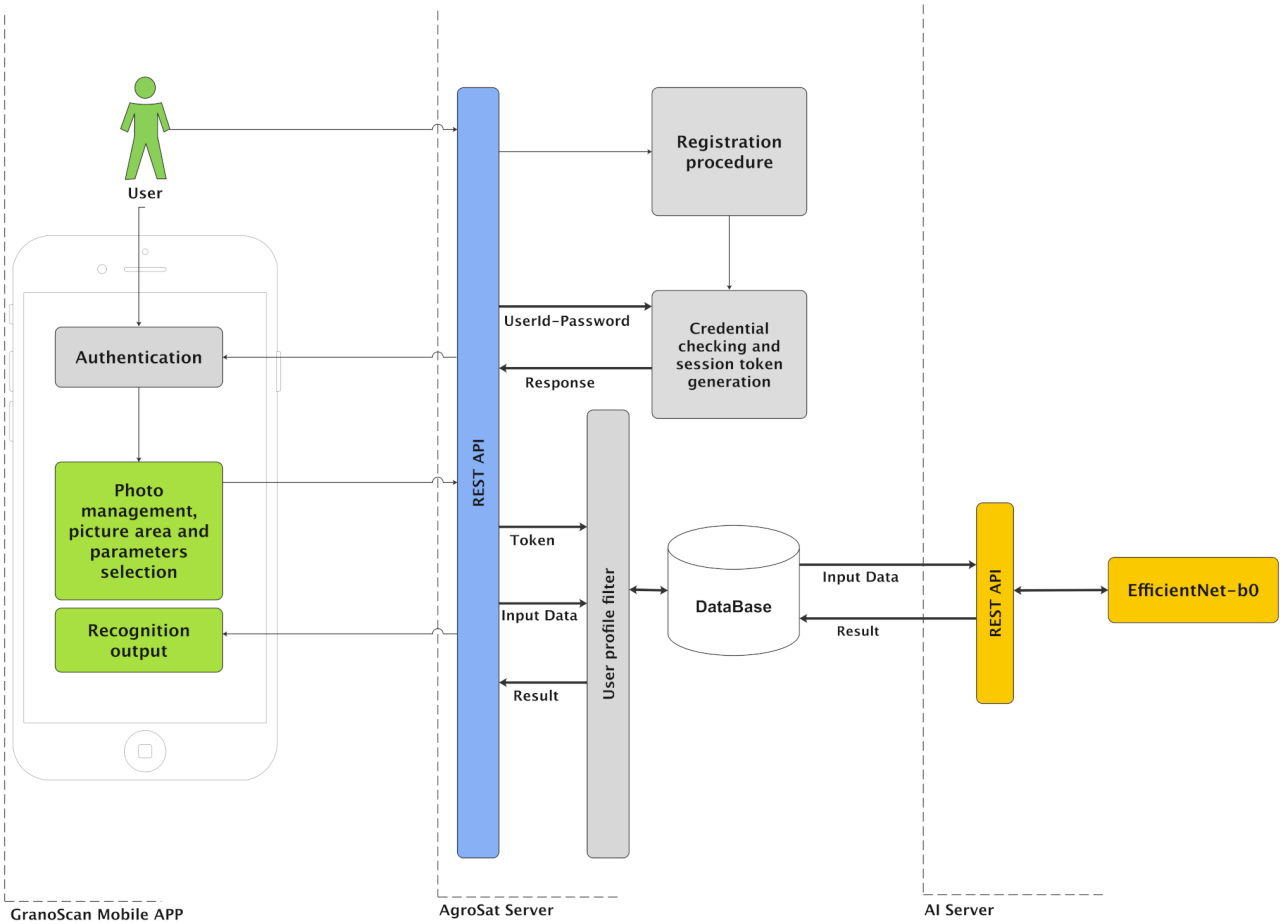
GranoScan internal architecture and data flows.

The deep learning model runs on a dedicated server that is not reachable by the mobile app directly. Interactions between the mobile app and deep learning server are managed by AgroSat APIs that receive data and requests by the mobile app, apply pre-processing activities, send data to the deep learning server, wait for results, store and send them back to GranoScan. **Figure 2** shows the internal architecture of the proposed solution, highlighting data flows among the GranoScan mobile app, AgroSat server and AI server.

## 3 Results

### 3.1 GranoScan co-design

**Figure 3:**
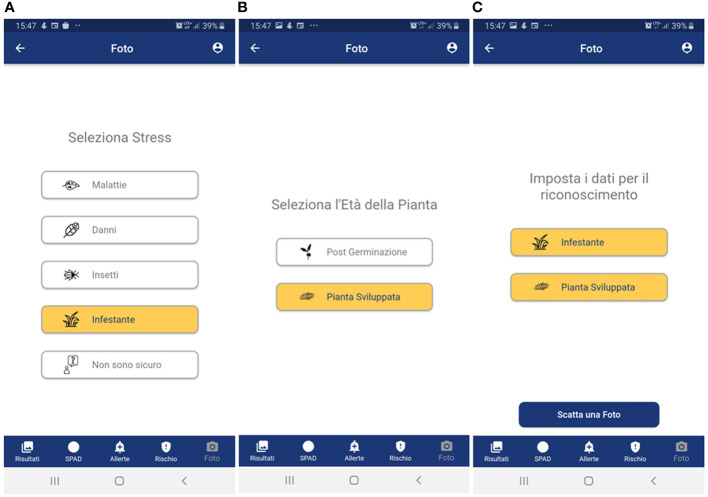
User-side walkthrough menu for wheat threat selection. In the example, a weed in the pre-flowering stage is selected through the following steps; **(A)** type of threat; **(B)** weed growth stage; and **(C)** summary of the selection choices before photo acquisition. Panel **A**: Seleziona stress = Select threat; Malattie = Disease; Danni = Damage; Insetti = Pests; Infestante = Weeds; Non sono sicuro = I am not sure. Panel **(B)**:Seleziona l’Età della Pianta = Select plant stage; Post Germinazione = Post-germination; Pianta Sviluppata = Developed plant; Panel **(C)**: Imposta i dati per il riconoscimento = Set the data for recognition; Infestante = Weeds; Pianta Sviluppata = Developed plant; Scatta una foto = Take a picture. For all panels: Foto = Photo; Risultati = Results; SPAD = SPAD; Allerte = Alerts; Rischio = Risk.

**Figure 4:**
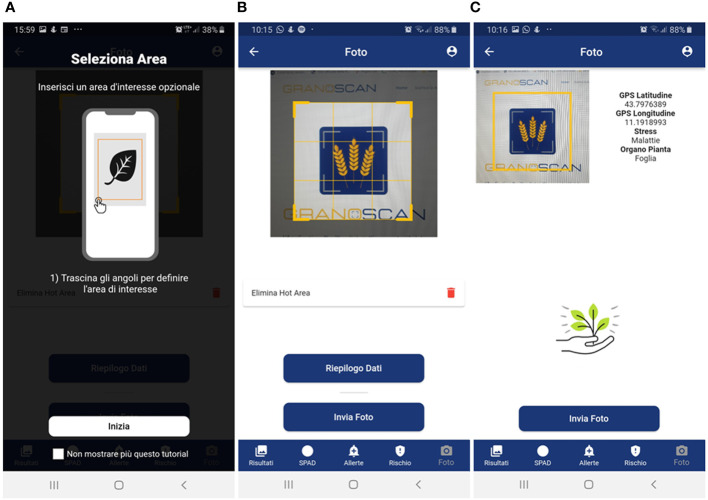
Mobile app photo acquisition steps: **(A)** brief guide for acquiring photos through users’ smartphone; **(B)** possibility of drawing an area of interest; **(C)** summary data – GPS coordinates, type of threat, plant organ – before sending the photo to the recognition system. Panel **(A)**: Seleziona Area = Select Area; Inserisci un’area di interesse opzionale = Select an optional area of interest; Trascina gli angoli per definire l’area di interesse = Drag the corners to define the area of interest; Inizia = Start; Non mostrare più questo tutorial = Don’t show this tutorial again. Panel **(B)**: Foto = Photo; Elimina Hot Area = Delete Hot Area; Riepilogo Dati = Data summary; Invia Foto = Send Photo; Risultati = Results; SPAD = SPAD; Allerte = Alerts; Rischio = Risk. Panel **(C)**: Stress = Threat; Malattie = Disease; Organo Pianta = Plant organ; Invia Foto = Send Photo; Risultati = Results; SPAD = SPAD; Allerte = Alerts; Rischio = Risk.

**Figure 5:**
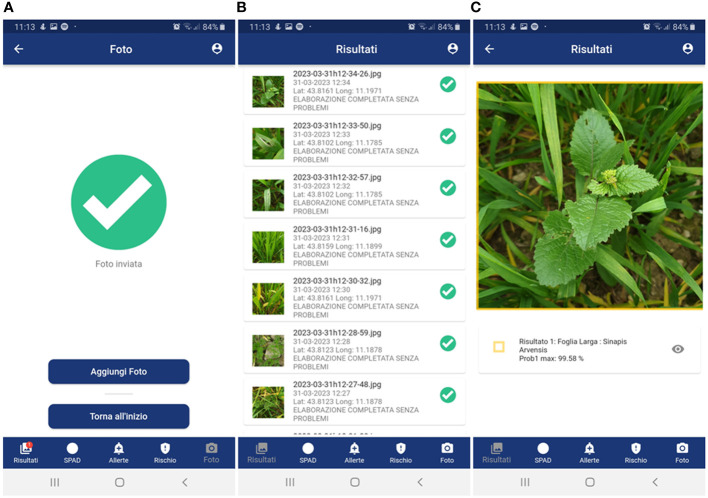
GranoScan recognition results: **(A)** notification of successful threat recognition; **(B)** list of all results; **(C)** detailed results (species and probability) of the target threat (in the example, weed in the pre-flowering stage). Panel **(A)**: Foto Inviata = Phot Sent; Aggiungi Foto = Add Photo; Torna all’inzio = Go back to the menu; Risultati = Results; SPAD = SPAD; Allerte = Alerts; Rischio = Risk. Panel **(B)**: Risultati = Results; Elaborazione completata senza problemi = Photo analyzed without any issues; Risultati = Results; SPAD = SPAD; Allerte = Alerts; Rischio = Risk. Panel **(C)**: Risultati = Results; Foglia larga = Dicot; Risultati = Results; SPAD = SPAD; Allerte = Alerts; Rischio = Risk.

**Figure 6:**
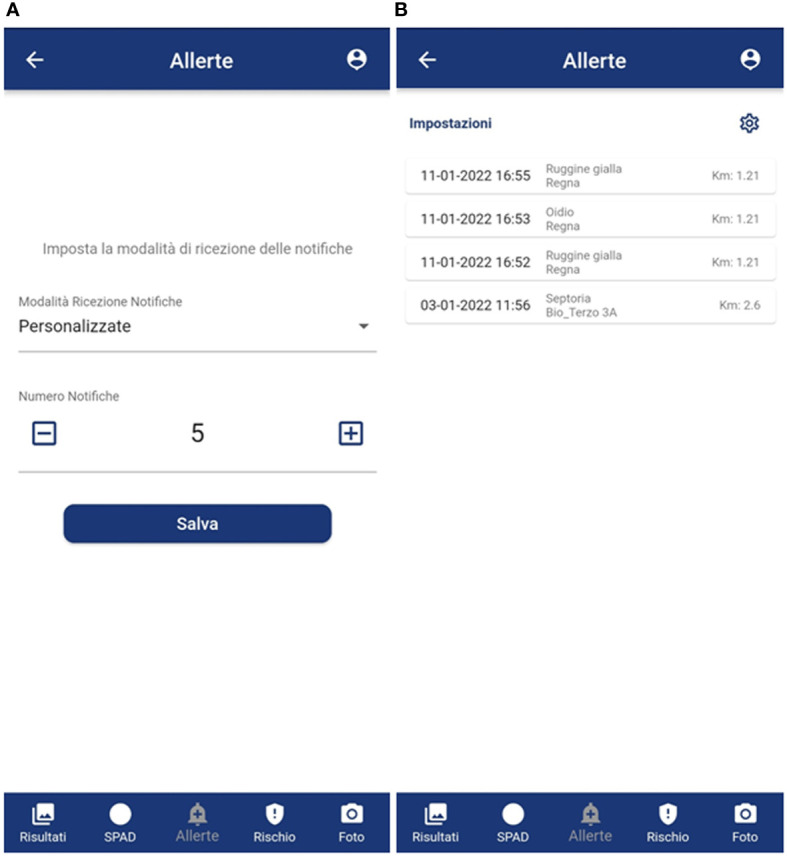
GranoScan alert tabs: **(A)** frequency setting of incoming notifications; **(B)** anonymous notifications of disease recognition in nearby fields (< 5 km), reporting date and time, type of threat and distance. Panel **(A)**: Allerte = Alerts; Imposta la modalità di ricezione delle notifiche = Set notification mode; Modalità Ricezione Notifiche = Notification receiving mode; Personalizzate = *Customized*; *Numero* notifiche = Notification number; Salva = Save; Risultati = Results; SPAD = SPAD; Allerte = Alerts; Rischio = Risk. Panel **(B)**: Allerte = Alerts; Impostazioni = Settings; Ruggine gialla = Puccinia s.; Oidio = Blumeria; Septoria = Septoria; Risultati = Results; Foglia larga = Dicot; Risultati = Results; SPAD = SPAD; Allerte = Alerts; Rischio = Risk.

The co-development process began in December 2019, with an initial exploration of the information needs and challenges faced by farmers in identifying insects, weeds and diseases, as well as evaluating farmers’ readiness to use phone-based digital tools. Among 40 participants, 80% identified the experience constraint (lack of references/knowledge) as the major constraint in the wheat threats recognition and reporting as clear examples of what usually happens in the recognition of weeds in post-germination and the experience that took place in previous years when the spikelets were damaged by late frost. The remaining 20% identified the major constraint in the timeliness of recognition and then receiving technical support. Among the 40 participants, a mismatch between the expected skills in using smartphone-based tools and the real ones clearly emerged, even just in the use of the camera and its settings. That said, the application was designed considering the following requests:

- to have a simple layout in terms of color, text and icons with the adoption of colors that can make use of the app simple in the disparate light conditions that can be encountered in the field (**Figures 3**–**6**) to provide, step-by-step, a brief guide to how the app works, which can be viewed or skipped;
- to provide a simplified menu to select the target (disease, weed, insect, damage, plant stage and plant organ) to photograph and make available the possibility of choosing the “I am not sure “ case where the user is not able to select the target to photograph (**Figure 3**);
- to optimize the use of the camera automatically and make camera parameter adjustment options available;
- after taking the photo, give the possibility to draw or not an area of interest to pay attention to for recognition (**Figure 4A**); otherwise, the central area of the image is selected (**Figure 4B**);
- to provide a summary of what was selected, and the photo taken before sending it for recognition (**Figure 4C**);
- to receive notification of the result as soon as it is available (**Figure 5A**), as well as always have all the results available in a dedicated tab (**Figure 5B**);
- to show the recognition results in decreasing order of accuracy (with a minimum threshold of 40%) for the image classification models and up to a maximum of 3 results (top 3);
- to show all the recognition results in decreasing order of accuracy (with a minimum threshold of 30%) for the image object detection models;
- to turn recognition results on the photo on or off (**Figure 5C**);
- to have the possibility to delete images from the results tab.

Furthermore, the exact previous percentages also emerged in indicating as fundamental: (i) the application must always be ready and running while working in the field (80% of the farmers), (ii) be informed in advance of the risk of disease to schedule field visit (20% of the farmers). In this regard, the application was designed with the possibility of working offline (without network coverage), enabling all menus, taking a photo (max 5 offline) and leaving everything in the buffer until the smartphone is hooked up to the net again to send the photo and receive the model output. At the same time, both to meet secondary needs and to create a community always updated, the possibility of receiving an anonymous notification relating to disease recognitions near one’s field (within 5 km) has been implemented (**Figures 6A, B**).

### 3.2 Deep learning results

**Figure 7:**
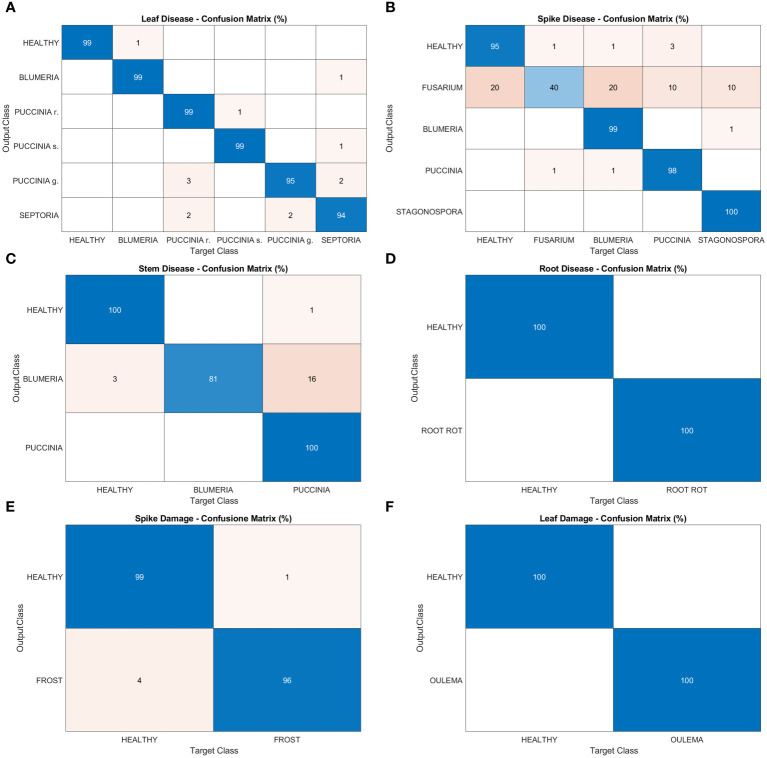
Confusion matrices of the deep learning architecture results for object detection models: **(A)** leaf disease, **(B)** spike disease, **(C)** stem disease, **(D)** root disease, **(E)** spike damage and **(F)** leaf damage.

In this section, the results regarding the performances of the deep learning architecture are reported. **Figure 7** shows confusion matrices for leaf disease, spike disease, stem disease, root disease, spike damage and leaf damage, respectively (panels A-F). Overall, a very good precision is reached in this phase, with peaks of 100% in leaf damage and root disease tasks and in some classes of spike and stem disease tasks. A precision of 99% is gained in the leaf disease task in every class excluding *Puccinia g.* (95%) and *Septoria* (94%). In the first case, the algorithm wrongly identifies as *Puccinia g.* leaves affected by *Puccinia r.* and *Septoria*; in the latter, *Blumeria g.*, *Puccinia r.* and *Puccinia g.* are confused with *Septoria*. The spike disease task presents the most inhomogeneous results among the object detection models. Indeed, alongside precision percentages that nearly achieve (99% and 98% for *Blumeria* and *Puccinia g.*, respectively) or reach the maximum (100% *Stagonospora*), there is a small portion of diseased spikes (5%) misrecognized as healthy. Fusarium head blight class has a weak performance, with a precision of 40%. The algorithm misclassifies as *Fusarium* all the other classes of the task: 20% of the analyzed regions of interest are actually healthy spikes, 20% are *Blumeria*, 10% *Puccinia* and 10% *Stagonospora*. For spike damages, a precision of 96% is reached in recognizing the negative impact of frost on wheat spikes, while the algorithm mistook mostly *Puccinia g.* for *Blumeria g.* (81% precision) in the stem disease tasks.

**Figure 8:**
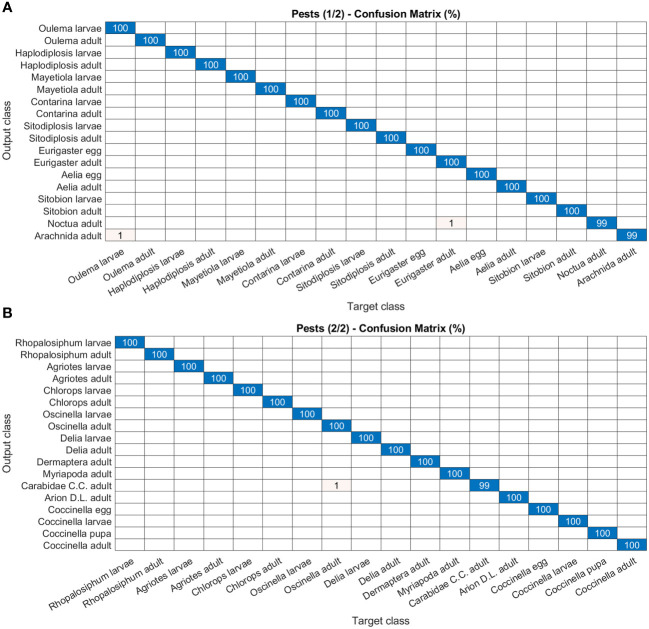
Confusion matrices of the deep learning architecture results for image classification models: **(A, B)** pests. The figure is split into two different panels to increase readability.

**Figure 9:**
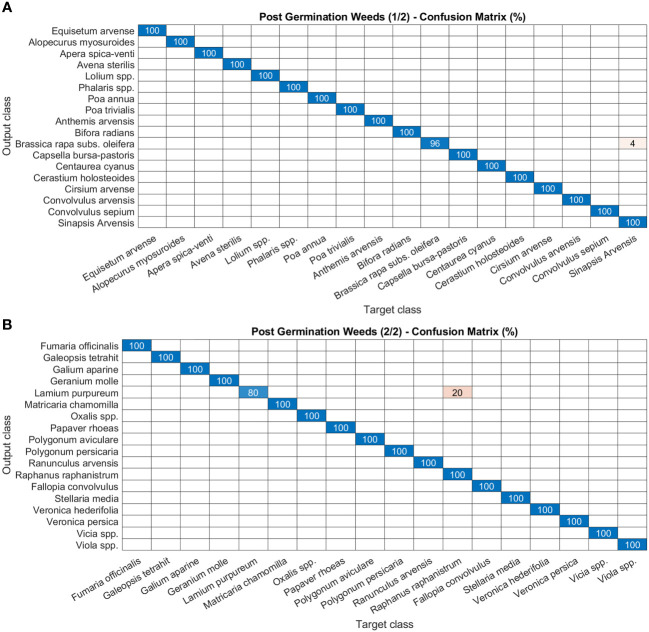
Confusion matrices of the deep learning architecture results for image classification models: **(A, B)** post-germination weeds. The figure is split into two different panels to increase readability.

**Figure 10:**
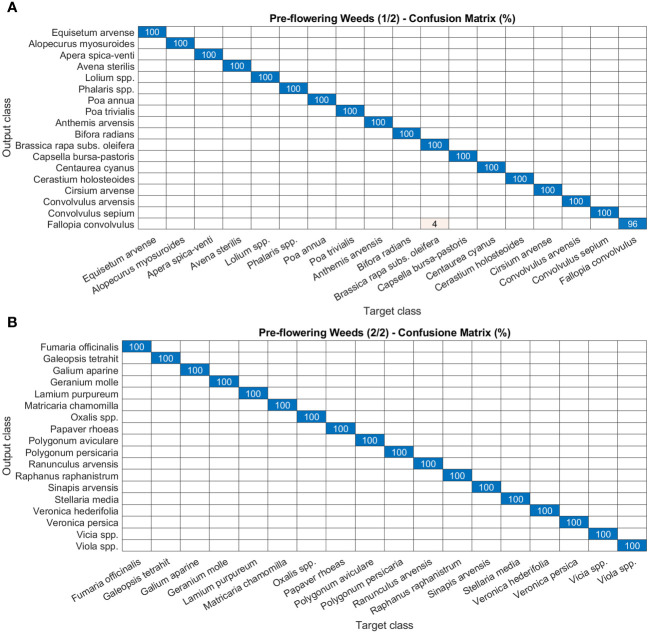
Confusion matrices of the deep learning architecture results for image classification models: **(A, B)** pre-flowering weeds. The figure is split into two different panels to increase readability.

As for disease and damage tasks, pests and weeds, for the latter in both the post-germination and the pre-flowering stages, show very high precision values of the models (**Figures 8**–**10**). In particular, most of the classes in the pest task report a precision of 100% and only three a slightly lower value (99%) (**Figures 8A, B**). For weeds in the post-germination phase, the trend in the precision values is similar to that observed for pests but there are two classes not reaching the top value. *Sinapis arvensis* is misclassified as *Brassica rapa* (96%). In addition, *Raphanus raphanistrum* is wrongly recognized by the models as *Lamium purpureum* (80%) (**Figures 9A, B**). Instead, a precision value of 100% in all the classes for pre-flowering weeds is gained, except in one case (96% precision) (**Figures 10A, B**).

**Figure 11:**
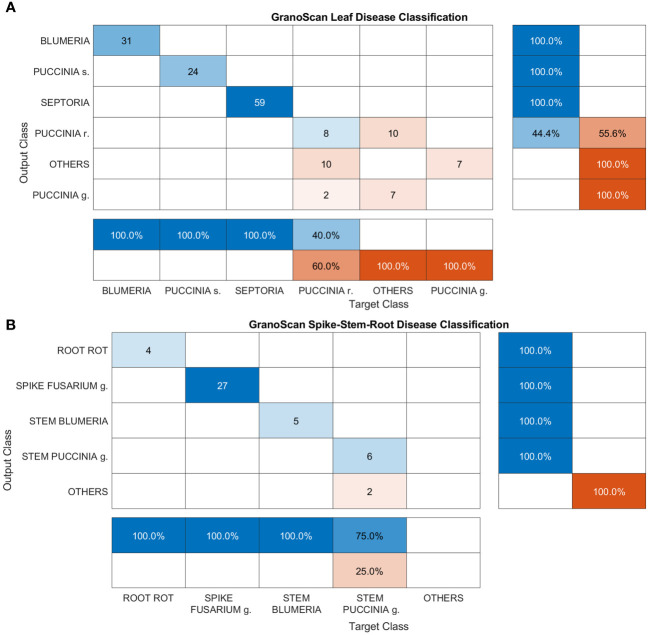
Confusion matrices of GranoScan in-field performances: **(A)** leaf disease and **(B)** spike, stem and root disease. For leaf disease classification the macro-average precision is 0.574 while the macro-average recall is 0.567. For spike-stem-root classification, the macro-average precision is 0.8 while the macro-average recall is 0.75.

### 3.3 GranoScan performances towards users’ real use

This section reports the performance of the mobile app in detecting and recognizing wheat threats directly in the field. Images were acquired by GranoScan users in the 2022 growing season (photos from March to July). In **Figure 11A**, classification results for leaf diseases are shown. *Blumeria*, *Puccinia s.* and *Septoria* are perfectly recognized by the system (100% precision and recall). This is due both to the high number of images used in the deep learning architecture development phase for these diseases and the good amount of target objects provided by the users (equal to 114). Regarding *Puccinia r.*, on one side the system shows a precision of 40%, inasmuch as it recognizes *Puccinia g.* and other threats as *Puccinia r.*; on the other, in some cases, *Puccinia r.* is not detected and is confused with other threats affecting wheat (recall 44.4%). Among leaf diseases, *Puccinia g.* has the worst performance: the system is not able to recognize it when actually present on the wheat leaf. Indeed, it is misclassified as *Puccinia r.* and other threats; in addition, other threats are wrongly classified as *Puccinia g.* Considering the lower occurrence of this disease with respect to other wheat rusts and the unfavorable climatic conditions for the fungus growth in spring 2022 in the Italian area, a limited number of users’ photos (9) were collected with initial disease symptoms. This could explain such a result in recognizing *Puccinia g.* In some cases, the AI tool classifies small, flat and non-dusty dark spots caused by other fungi or physiological deficiencies (e.g. micro-nutrients), which are not encompassed in the GranoScan wheat threat list, as brown (*Puccinia r.*) or black rust (*Puccinia g.*). Overall, for the leaf disease classification task, the macro-average precision is 0.574 while the macro-average recall is 0.567; the mean accuracy of the system results of about 77%.

Spike, stem and root diseases have been grouped and classification results are reported in **Figure 11B**. For these three tasks, the images acquired by GranoScan thanks to the users’ field activity make it possible to obtain a fairly limited number of recognition responses (44). Nevertheless, the overall accuracy of the AI tool is high, yielding a value of 95%. Root rot, *Fusarium* head blight and stem powdery mildew are correctly classified by the system. Only *Puccinia g.* on the stem doesn’t reach the top accuracy but still reports a good precision value (75%). In particular, in two cases, other threats affecting wheat are misclassified as stem black rust. For spike-stem-root classification, the macro-average precision is 0.8 while the macro-average recall is 0.75.

Regarding the pest classification task, the app returns the top 3 results (see section 3.1). The top 1 classification result has an overall accuracy of 80% while the top 3 reaches a value of 94%, i.e. the first response of the system is always correct in 80% of cases and the right recognition result is provided by the top 3 in 94% of cases, respectively (data not shown).

For weeds, GranoScan shows a great ability (100% accuracy) in recognizing whether the target weed is a dicot or monocot in both the post-germination and pre-flowering stages while it gains an accuracy of 60% for distinguishing species. The latter performance is negatively affected by some users’ photos capturing weeds which are not encompassed in the GranoScan wheat threat list and therefore not classified by the proposed models (data not shown).

## 4 Discussion

GranoScan (GranoScan, 2023) is the first free mobile app dedicated to the in-field detection and recognition of over 80 threats (diseases, pests, weeds, biotic/abiotic damages) affecting wheat. GranoScan, available in the main online stores, is aimed at all users of the wheat supply chain to provide support in the localization and recognition of the main threats directly in the field. Potential users are represented by agronomists, consultants and elevators, but the app is mainly addressed to farmers. Embracing the idea that there is a need to involve the potential users of the tool under design in the design processes (Barcellini et al., 2022), we adopted a co-design approach involving a group of farmers. Co-design is a process to rapidly develop technologies better matched to user needs (McCampbell et al., 2022) and seeks to build and maintain a shared conception of the design problem to allow collaboration (Gardien et al., 2014). By involving heterogeneous stakeholders in the collective exploration of solutions to a common problem, we sought to overcome the linear model reported by Berthet et al (Berthet et al., 2018). consisting of scientific and technical knowledge produced in research organizations, further development of technologies carried out through public and private technical institutes that disseminate innovation to farmers, being the end-users. As recommended by Eastwood et al. (Eastwood et al., 2022), we engaged with farmers early in the problem definition stage and the development of the app’s initial prototype. Then, we evolved the co-design process into a second phase involving ICT experts to further develop prototype concepts; finally, we re-engaged farmers in testing. This workflow allows to tackle some of the main barriers constraining ICT adoption by farmers, such as inadequate computer skills, unawareness of the potential of ICT solutions to contribute to the farm business and access to broadband in rural areas (Wims and Byrne, 2015).

In the first phase, we held monthly meetings to discuss the app’s purpose and functionality and to gather feedback on the app’s features and use. Farmers expressed ideas on what a profitable mobile app would look like and mentioned design features such as simplicity, user-friendliness, offline options, tutorial boxes and data security measures (e.g. log-in procedure). Careful development of the application interface in terms of visual aesthetics is important (Mendes et al., 2020), as it is usually the first characteristic that a user notices when downloading an application and in turn could affect the functionality and usability of the app (Siddiqua et al., 2022). We discussed with farmers app graphic features, such as colors, icons and text size, also evaluating their appropriateness to the different light conditions that can occur in the field. Also buttons, icons and menus on the screen were designed to ensure an easy user navigation between components and an intuitive interaction between components, with a quick selection from a pre-set menu. To ensure the usability of GranoScan also with poor connectivity or no connection conditions affecting rural areas in some cases, the app allows up to 5 photos to be taken, which are automatically transmitted as soon as the network is available again. Once the photo upload is complete, the implemented synchronization system allows new shots both online and offline. Farmers also expressed the need to be informed of any plant diseases found in fields close to their own. For this purpose, an alert system was developed exploiting the smartphone push notifications that remind users of the app feature and improve the app’s usage frequency. Finally, farmers were involved in the early stages of GranoScan implementation starting from the aesthetics and functionality to the technical content regarding crop protection. In this sense, they represented a source of advice and a term of comparison for selecting the most widespread and threatening diseases, pests and weeds affecting wheat in the Italian area.

In the second phase of the co-design process, after the first prototype release, the farmers involved were asked to test the app respecting their real working conditions (early prototype testing) (Prost, 2021) and provide further feedback to adjust and refine the design. When the final prototype was completed, the first group of farmers was involved in the prototype promotion towards a bigger group of farmers (peer-to-peer activity). The task was designed this way since farmers represent a category of practitioners who prefer peer-to-peer learning and are experiential learners (Sewell et al., 2017). We followed and embraced this co-design approach because it is crucial to design new technologies jointly with farmers in a participatory manner rather than imposing them and expecting end users to adopt and adapt (Kenny and Regan, 2021).

Regarding the performances of AI tool model development, the results show a very positive trend with high levels of precision. The proposed AI models are, therefore, certainly a key component and a central contribution of the paper; yet, their innovative points rely not only on the introduction of an innovative deep learning approach capable of addressing plant science problems but mainly on the effective training of such models and their integration in an operative service thanks to the proposed mobile app for in-field identification of wheat threats. It should be noted that a few classification tasks could be improved, as for *Blumeria g.* among the stem diseases. In this case, the dimmed light conditions of images acquired in the lower part of the stem and the similarity of symptoms (black spot) between *Puccinia g.* and *Blumeria g.* in the later growth stages could represent the main reasons for this misclassification. In addition, *Fusarium* head blight in the spike disease task shows the lower precision of the dataset. This could be mainly due to many dataset images with the co-occurrence of a high number of spikes and varied coloring of spike and fungal bodies shifting from wheat flowering, post-flowering till harvest stage. For the weed classification task, only two species (*Brassica rapa* and *Lamium purpureum*) don’t reach the top value of precision in the post-emergence stage. The misclassification could be explained by the similarity of the seedlings (in the case of *Brassica rapa* vs. *Sinapis arvensis* both species belong to the *Brassicaceae* family), and above all by the small dimensions (often < 2 cm) of the target objects in the images, where plant details are hard to distinguish.

Regarding recognition accuracy towards end-users’ in-field photos, GranoScan achieved very good performances, overall. Our results conform to or outperform those of other studies deploying AI models on mobile devices. It is worth noting that there is a lack of scientific works dealing with this topic that validate their results through an external image dataset, as is done in this study (see section 3.3). So, the comparison of the results is somewhat hindered. For leaf diseases, recognition performances are excellent (100% accuracy for powdery mildew, *Septoria* and yellow rust), except for brown rust (44.4% accuracy). Johannes et al. (Johannes et al., 2017). reported accuracy values for septoria and rusts (calculated for yellow and brown rust together) of 79% and 81%, respectively while Picon et al. (Picon et al., 2019). (which extended the previous work) improved model performance by gaining an accuracy of 96% for *Septoria* and 98% for rusts. In both studies, the results were validated under real conditions, in different study sites. Performing a disease and non-disease classification for wheat yellow rust, Tang et al. (Tang et al., 2023). achieved accuracies ranging from 79% to 86% by independently validating the system on a published dataset from Germany. Therefore, considering the mean accuracy for the two classes of yellow and brown rust (76%), our results are in line with the cited papers, outperforming *Septoria* while gaining slightly lower results for rusts. On the other hand, the system is not able to correctly classify images from users framing black rust. This could be due to the limited amount of original training images (120 for leaf black rust). As for other classes, data augmentation, which provides a promising means to address the insufficiency of collected images, is used here to algorithmically expand the scale of the dataset. However, it seems that the main reason for such a performance could also be the limited number of images from users (only 9) during 2022. In this sense, a new deep learning approach dealing with small sample-size datasets, such as that presented by Liu and Zhang (Liu and Zhang, 2023), is demonstrating effectiveness and feasibility in disease classification tasks. Diseases affecting other wheat organs have excellent classification performances; only black rust on the stem presents a slightly lower value.

The system gains very good performances also in recognizing pests (80 and 94% top 1 and top 3 accuracies, respectively), with slightly lower results with respect to Karar et al. (Karar et al., 2021). This study presents a classification accuracy of 98.9% on five groups of pests (aphids, *Cicadellidae*, flax budworm, flea beetles and red spider) but without validating the AI model through an external dataset. Regarding weed recognition, GranoScan obtains excellent results (100% accuracy) in distinguishing if a weed is a monocot or a dicot, while it reaches an accuracy of 60% in species classification. In the first case, our results outperform other studies (Teimouri et al., 2022) while in the second present a slightly lower value (e.g. 77% for Madsen et al. (Madsen et al., 2020) gained by processing the images with a workstation and without evaluating the AI tool through an external dataset). These performances in weed recognition are mainly due to the high number of training images for target species. It is worth noting that the most essential building block for an AI model is the underlying data used to train it (Sharma et al., 2020). In addition, enabling computer vision for precision agriculture requires vast (e.g. tens of thousands of images) and specialized datasets, especially collected under a realistic environment, to account for a wide range of field conditions (Lu and Young, 2020). In this sense, the AI model for weed classification task in GranoScan benefits from an in-house image dataset built through a long phenotyping activity. In the framework of precision agriculture, interest in the early management of weeds, knowing if they are dicots or monocots, makes our results very valuable for final users. Identifying whether the target plant is a grass or broadleaf weed provides crucial information for management strategies, such as active ingredients for chemical control. Thus, pushing the recognition down to the species detail may not be so determining (Dainelli et al., 2023).

Looking at the few unsatisfactory performances of GranoScan, we are conscious that troubleshooting is not straightforward. Indeed, most AI models for automatic diseases, pests and weeds recognition suffer from reduced performance when applied to real environment images previously unseen (Sharma et al., 2020). The main reasons are: (i) many discriminative details and features of crop threats are small, blurred, hidden and lacking in details, making the targets hard to distinguish from the background; (ii) the diversity and complexity of scenes in the field cause a variety of challenges, including dense or sparse distribution, illumination variations and occlusion (Patrício and Rieder, 2018; Wang et al., 2021).

Briefly comparing GranoScan on recognition features towards other diagnostic apps, which are supported by scientific articles and listed in the Introduction section, these are the main outcomes. ApeX−Vigne (Pichon et al., 2021) monitors water status using crowdsourcing data but is dedicated to grapevine and hence is not suitable for a proper comparison. BioLeaf (MaChado et al., 2016) measures only foliar damage caused by insects, estimating the percentage of foliar surface disrupted (% defoliation); it encompasses neither insect species recognition nor other categories of threats. PlantVillage Nuru (Coletta et al., 2022), leveraging a crowdsensing platform, performs disease diagnosis in developing countries for several plant species; in the crop list, there is wheat but currently diseases affecting this crop are not recognized and the app works only in survey mode for images acquisition. PlantifyAI (Shrimali, 2021) is developed for diagnosing diseases across several crop species, including wheat, and offers also control methods; unfortunately, the diagnosis tool for disease recognition is available only by paying a weekly/annual fee. Plantix (Tibbetts, 2018) detects diseases, pests, and nutritional deficiencies in 30 crops, including wheat; the app is well organized and the graphic interface is user-friendly. The app has also an alert tool for pests and diseases. However, by testing the app on wheat diseases, the recognition results are not always in accordance with the target and, in complex images (i.e. occluded and with dense vegetation), often the output results as “unknown disease detected”. Besides, no weed recognition is provided. In this framework, to continuously optimize the proposed app, future work will be dedicated to comparing GranoScan with other agricultural apps not included in the current research.

GranoScan was officially released in spring 2022, so our results take into account only one growing season (image data from users of the 2023 wheat growing season are not included in this study). We are confident in better future performances since AI model updates are scheduled and a growing amount of in-field images is expected. In this sense, after a supervision process conducted by crop science researchers for all the incoming images, the new photos will enrich the training dataset. This way, the expanding dataset thanks to user activity and the self-learning techniques on which the app is based will allow GranoScan to gain continuously improving results.

GranoScan is an evolving tool and future improvements will include the AI model update (also switching from label to pixel classification to optimize the recognition of critical diseases, such as *Puccinia r.*), the enrichment of training image dataset drawing also from external sources, translating the app interface into other languages to allow its use in the entire Mediterranean area and, following the co-design approach, the extension of the recognition task to new wheat threats thanks to user feedback. Besides, the already ongoing data trade-off services, such as the geolocation of acquired images, between the web platform AgroSat (AgroSat, 2023) and GranoScan will be boosted.

## 5 Conclusions

This research presents the development and first results of GranoScan, a mobile app for localization and in-field recognition of the main threats affecting wheat based on an ensembling strategy that uses two instances of the EfficientNet-b0 architecture as core models. It is one of the first mobile apps available for free in the main online stores created within a research project. GranoScan is addressed to field users, particularly farmers, which contributed to the app implementation through a co-design approach.

The idea and the development of GranoScan stand from the necessity to give to the wheat chain stakeholders (mainly farmers and technicians) a digital tool free, easy to use and always accessible. To the best of our knowledge, a mobile app specifically dedicated to the recognition of wheat abiotic and biotic stresses, supported by a public scientific activity and co-designed together with end users, is lacking. GranoScan is based on a large dataset (almost 70000 images) due to the need for robust training and validation of AI models, especially when the tool is dedicated to outdoor recognition activity. In this sense, every time threat identification is a challenge considering changes in light, climate conditions and phenotypic expressions of wheat varieties that can affect how a threat arises. Tackling these issues, the study contributes to generating a new deep learning architecture gaining recognition performances equal to or better than other similar mobile applications. To fill the gap between the positive attitude toward a new agricultural app and the negative usage level as experienced by other studies, an original co-design approach was used throughout the implementation process of the app, from the collection of user needs to the choice of operative solutions and system debugging. As one of the major contributions of the study, the research activity managed to establish successfully a trained user community, able to promote and spread the GranoScan app among other farmers.

Within this framework, the usefulness of GranoScan can be summarized as follows:

- to improve user skills in recognizing uncommon threats affecting wheat;
- to facilitate the user in requesting technical advice in the field, through support on threat recognition;
- to allow the in-field geolocation of threats, to facilitate new inspections and/or verify the effectiveness of phytosanitary treatments;
- to promote tools (risk model and early warning) that allow a timely management plan to ensure economic and environmental sustainability;
- to create a community of farmers always updated about the threat pressure near their fields.

Two crucial factors emerge from this study that can support future development of agricultural apps: (i) the importance of adopting a user-centered design to enhance the capacity of all farmers to participate in, contribute to, and benefit from agricultural innovation development; (ii) the engagement of farmers from the initial stages of tool implementation turns out to be a win-win solution. The first element proved to be a guarantee of achievement of an app that is simple and effortless to use, accessible to and understood by all farmers; the second one unleashed farmers in involving other farmers and this increased the source of information (photos) used for training our AI models. Indeed, one of the biggest challenges in solving agricultural problems using artificial intelligence approaches is the lack of available large datasets from field conditions. As potential expansions, more wheat threats will be included in GranoScan functionalities as well as the translation of the app in multilanguage to assist farmers in the whole Mediterranean area further.

## Data availability statement

The original contributions presented in the study are publicly available (see the weed phenotyping image dataset). This data can be found here: https://doi.org/10.5281/zenodo.7598372.

## Author contributions

RD: Conceptualization, Data curation, Investigation, Methodology, Visualization, Writing – original draft, Writing – review & editing. AB: Data curation, Formal analysis, Methodology, Software, Writing – original draft. MM: Data curation, Formal analysis, Methodology, Software, Writing – original draft. DM: Data curation, Formal analysis, Methodology, Software, Writing – original draft. LR: Software, Writing – review & editing. SM: Writing – review & editing. EF: Writing – review & editing. MS: Writing – review & editing. SA: Writing – review & editing. PL: Writing – review & editing. PT: Conceptualization, Data curation, Formal analysis, Funding acquisition, Methodology, Project administration, Supervision, Validation, Visualization, Writing – original draft, Writing – review & editing.
