# Supplementary material for: GranoScan: an AI-powered mobile app for in-field identification of biotic threats of wheat
Source: Front Plant Sci. 2024 Jun 7;15:1298791. doi: 10.3389/fpls.2024.1298791 (PMC11190326; doi:10.3389/fpls.2024.1298791)
Supplement: Supplementary file 1 [file Table_1.docx]

Supplementary Material

# Supplementary Tables

**Table S1**. Fungal diseases: scientific name and affected plant organs.

| **Fungal disease** | | **Scientific name** | **Plant organ** |
| --- | --- | --- | --- |
| Wheat powdery mildew | | *Blumeria graminis f. sp. tritici* | Leaf, stem, spike |
| Brown rust | | *Puccinia recondita f.sp.tritici* | Leaf |
| Yellow rust | | *Puccinia striiformis f. sp. tritici* | Leaf |
| Black rust | | *Puccinia graminis f.sp.tritici* | Leaf, stem, spike |
| Septoria | Septoria tritici blotch | *Septoria tritici* | Leaf |
|  | Septoria nodorum blotch | *Stagonospora nodorum* | Spike |
| Fusarium head blight (FHB) | | *Fusarium graminearum* | Spike |
| Root rot | Dryland root rot | *Fusarium spp.* | Root |
|  | Common root rot | *Bipolaris sorokiniana* | Root |
|  | Take-all root rot | *Gaeumannomyces graminis* | Root |

**Table S2**. Pests: scientific name and life cycle stages

| **Pests** | **Scientific name** | **Life cycle stage** |
| --- | --- | --- |
| Cereal leaf beetle | *Oulema melanopus* | Larvae, adult |
| Saddle gall midge | *Haplodiplosis marginata* | Larvae, adult |
| Hessian fly | *Mayetiola destructor* | Larvae, adult |
| Wheat fly | *Contarina tritici* | Larvae, adult |
| Wheat midge | *Sitodiplosis mosellana* | Larvae, adult |
| Tortoise bug | *Eurygaster maura* | Egg, adult |
| Shield bug | *Aelia rostrata* | Egg, adult |
| English grain aphid | *Sitobion avenae* | Larvae, adult |
| Bird cherry-oat aphid | *Rhopalosiphum padi* | Larvae, adult |
| Click beetles * | *Agriotes* | Larvae, adult |
| Chloropid gout fly | *Chlorops pumilionis* | Larvae, adult |
| Frit fly | *Oscinella frit* | Larvae, adult |
| Wheat-bulb flies | *Delia coarctata* | Larvae, adult |
| Earwigs * | *Dermaptera* | Adult |
| Myriapods * | *Myriapoda* | Adult |
| Ground beetles, leaf beetles, snout beetles * | *Carabidae*, *Chrysomelidae*, *Curculionidae* | Adult |
| Moths * | *Noctua* | Adult |
| Mites * | *Arachnida* | Adult |
| Land slugs * | *Arion*, *Deroceras*, *Limax* | Adult |
| Ladybird * | *Coccinella* | Egg, larvae, pupa and adult |

* The identification of these pests is carried out at the taxonomic level indicated in the table

**Table S3**. Weeds: botanical classification and scientific name.

| **Weed** | **Scientific name** |
| --- | --- |
| *Tracheophytes* | |
| Common horsetail | *Equisetum arvense* |
| *Monocot* | |
| Black grass | *Alopecurus myosuroides* |
| Common windgrass | *Apera spica venti* |
| Wild oat * | *Avena sterilis* |
| Rye-grass * | *Lolium spp.* |
| Canary grass | *Phalaris spp.* |
| Annual bluegrass | *Poa annua* |
| Rough bluegrass | *Poa trivialis* |
| *Dicot* | |
| Mayweed | *Anthemis arvensis* |
| Wild bishop | *Bifora radians* |
| Field mustard | *Brassica rapa subs. oleifera* |
| Shepherd's purse | *Capsella bursa-pastoris* |
| Cornflower | *Centaurea cyanus* |
| Common mouse-ear chickweed | *Cerastium holosteoides* |
| Creeping thistle | *Cirsium arvense* |
| Field bindweed * | *Convolvulus arvensis* |
| Hedge bindweed | *Convolvulus sepium* |
| Black-bindweed | *Fallopia convolvulus* |
| Common fumitory * | *Fumaria officinalis* |
| Common hemp-nettle | *Galeopsis tetrahit* |
| Cleavers | *Galium aparine* |
| Dovesfoot geranium | *Geranium molle* |
| Purple dead-nettle | *Lamium purpureum* |
| Wild chamomile | *Matricaria chamomilla* |
| Wood sorrel | *Oxalis spp.* |
| Common poppy * | *Papaver rhoeas* |
| Common knotgrass | *Polygonum aviculare* |
| Lady's thumb | *Polygonum persicaria* |
| Field buttercup | *Ranunculus arvensis* |
| Wild radish | *Raphanus raphanistrum* |
| Charlock mustard | *Sinapis arvensis* |
| Chickweed | *Stellaria media* |
| Ivy-leaved speedwell | *Veronica hederaefolia* |
| Common field-speedwell * | *Veronica persica* |
| Vetch * | *Vicia spp.* |
| Pansy | *Viola spp.* |

* Weed species subjected to phenotyping activity. For rye-grass and vetch, *Lolium multiflorum* and *Vicia sativa* are used for imaging, respectively.
